# S06-2 GAP: The Girls Active Project

**DOI:** 10.1093/eurpub/ckac093.029

**Published:** 2022-08-29

**Authors:** Sara McQuinn, Sarahjane Belton, Anthony Staines, Mary Rose Sweeney

**Affiliations:** 1 School of Nursing, Psychotherapy and Community Health, Faculty of Science and Health, Dublin City University, Dublin 9, Ireland; 2 School of Health and Human Performance, Faculty of Science and Health, Dublin City University, Glasnevin, Dublin 9, Ireland

**Keywords:** Adolescent females, school-based, intervention, intervention design, behaviour change wheel

## Abstract

**Background:**

Adolescent females Physical Activity (PA) participation rates are low globally, particularly among females of lower Socio-Economic Status (SES). Evidence suggests theory‐based, multi-component interventions are most effective at improving PA levels. This research aimed to co-design, with adolescent females, a theory-driven, multi-component, extracurricular school-based PA intervention, the Girls Active Project (GAP) and assess its feasibility.

**Setting:**

One single-sex, females-only, designated disadvantaged post-primary school in Dublin, Ireland.

**Methods:**

The Behaviour Change Wheel (BCW) and Public and Patient Involvement (PPI) were used to develop the GAP. Mixed-methods with students (n = 287, aged 12-18) and teachers (n = 7) captured students’ self-reported PA levels and identified factors influencing PA behaviour at school. These data were subsequently used in discussion groups with PPI contributors (n = 8, students aged 15-17) to co-design the intervention. Mixed-methods were applied with multiple stakeholders to assess the feasibility of implementing and evaluating the GAP programme over a 12-week single-arm feasibility trial.

**Results:**

Just 1.4% of the students in this sample (n = 287) reported meeting the recommended PA guidelines. Time, social influences, beliefs about capabilities, environmental context and resources, goals, reinforcement, and behavioural regulation emerged from the data as factors influencing PA behaviour. A peer-led, after-school PA programme was co-designed. The feasibility study encountered significant contextual barriers and challenges with recruitment. Recruitment (n = 8, 10%) was low, yet retention (88%) was high. Despite the COVID-19 pandemic hindering implementation, results suggested the GAP programme was implemented with high fidelity (87%), well-received by stakeholders and perceived as compatible with the after school-setting.

**Conclusions:**

PA levels of females in this sample were far below recommended guidelines for optimum health. The novel approach applied to systematically co-design the intervention could facilitate future replication. Whilst further thought must be given on how to increase enrolment, the in-person delivered PA programme showed promise as an intervention that can be feasibly implemented and evaluated. Future research should examine the GAP’s preliminary-effectiveness at increasing PA levels in a pilot-cluster randomised controlled trial.

